# Major dietary patterns in relation to chronic low back pain; a cross-sectional study from RaNCD cohort

**DOI:** 10.1186/s12937-022-00780-2

**Published:** 2022-05-12

**Authors:** Yahya Pasdar, Behrooz Hamzeh, Sheno Karimi, Shima Moradi, Sahar Cheshmeh, Mohammad Bagher Shamsi, Farid Najafi

**Affiliations:** 1 Department of Nutritional Sciences, Research Center for Environmental Determinants of Health (RCEDH), Health Institute, Kermanshah University of Medical Sciences, Kermanshah, Iran; 2 Environmental Determinates of Health Research Center, School of Public Health, Kermanshah University of Medical Sciences, Kermanshah, Iran; 3 Department of Community Nutrition, School of Nutrition and Food Sciences, Isfahan University of Medical Sciences, Isfahan, Iran; 4 School of Nutritional Sciences and Food Technology, Kermanshah University of Medical Sciences, Kermanshah, Iran; 5 Rehabilitation and Sports Medicine Department, Kermanshah University of Medical Sciences, Kermanshah, Iran; 6 School of Public Health, Communing Developmental and Health Promotion Research Center, Kermanshah University of Medical Sciences, Kermanshah, Iran

**Keywords:** Low back pain, Diet, Dietary pattern, High protein diet

## Abstract

**Background:**

Chronic low back pain (LBP) is the most common musculoskeletal pain that affects a person’s daily activities. This present study aimed at evaluating the relationship between major dietary pattern and Chronic LBP.

**Methods:**

This cross-sectional analysis was examined 7686 Kurdish adults. The RaNCD cohort study physician diagnosed chronic LBP. Dietary patterns were derived using principal component analysis. The three identified dietary patterns derived were named: 1) the vegetarian diet included vegetables, whole grain, legumes, nuts, olive, vegetable oil, fruits, and fruit juice; 2) high protein diet related to higher adherence to red and white meat, legumes, nuts, and egg; and 3) energy-dense diet characterized with higher intake of salt, sweet, dessert, hydrogenated fat, soft drink, refined grain, tea, and coffee. Dietary pattern scores were divided into tertiles. Binary logistic regression in crude, adjusted odds ratios (OR) and 95% confidence intervals (CI) were used to determine this association.

**Results:**

Twenty-two per cent of participants had chronic LBP. Higher adherence to high protein dietary pattern was inversely associated with chronic LBP in crude (OR: 0.79, 95% CI: 0.69–0.9) and adjusted model (for age, sex, smoking, drinking, diabetes, physical activity, body mass index, and waist circumference) (OR: 0.84, 95% CI: 0.72–0.97). In addition, after controlling for the mentioned potential confounders, participants in the highest category of energy dense diet were positively associated with chronic LBP compared with those in the lowest category (OR: 1.13, 95% CI: 1.01–1.32).

**Conclusions:**

Higher adherence to the high protein diet was inversely related to chronic LBP prevalence. In addition, we found that following energy dense diet was positively associated with chronic LBP.

## Introduction

Low back pain (LBP) is the main cause of disability in the United States, with more than 1 in 5 adults experiencing chronic pain [1, 2]. In global disease burden studies, LBP usually ranks first when disease burden is measured by disability and is also in the top ten if both death and disability are considered [3]. LBP is caused by problems related to the intervertebral discs, nerves, muscles, etc., in the lumbar and sacral vertebrae [4]. Most LBP patients (up to 90%) have non-specific pain without apparent cause [5]. LBP is classified into three categories based on the duration of symptoms. Acute LBP is often the result of actual or near tissue injury or sprain, which has been present for 6 weeks or less, and it tends to settle on its own with personal care. Sub-acute LBP has a six- to 12-week duration, and chronic LBP lasts longer than 12 weeks. According to this category, chronic LBP often persists even though the initial injury has healed. These cases are more likely to be referred for treatment than the more acute cases that linger untreated [6].

People with chronic LBP have difficulty in social and occupational activities. Even the resulting pain affects a person’s mood and puts a heavy burden on the treatment system; overall, chronic LBP is the most common cause of disability in a person’s daily life activities [7]. It should be noted that many people may not see a doctor and consider a self-medication approach, so its prevalence is higher in communities [8]. Evidence suggests that stress, anxiety, sedentary lifestyle, hard work, obesity, and diet are involved in the etiology of chronic LBP [9].

Increased levels of pro-inflammatory mediators in the body can be involved in the pathogenesis of chronic LBP [10, 11]. Adherence to an unhealthy diet pattern by producing pro-inflammatory mediators upsets the balance of these mediators in the body [12]. Higher adhere to the Western diet, which is characterized by higher intake of refined grains, red meat, processed meat, high saturated fat, trans-fatty acids, sweet sugary foods, and caffeine, as an unhealthy diet is associated with the production of high levels of cytokines, interleukins, C- reactive protein (CRP) and tumor necrosis factor α (TNF-α) [4, 13]. A review study has recently indicated that adherence to the Mediterranean and plant-based diet, associated with consuming vegetable oils, especially olive oil, effectively reduces musculoskeletal pain [14]. A healthy dietary pattern seems related to an adequate and balanced intake of all food groups that can moderate the inflammatory conditions of the body [15, 16].

The high prevalence of chronic LBP worldwide and the importance of proper diet in reducing inflammatory conditions necessitates studying the relationship between major dietary patterns and chronic LBP among the Kurdish population.

## Material and methods

### Study design and participants

This cross-sectional study was conducted on data from the recruitment phase of the Ravansar non-communicable diseases (RaNCD) cohort study. This population-based study was conducted amongst the Kurdish population (4770 men and 5289 women) aged 35–65 years residing in Ravansar, Kermanshah province, Western Iran. This study was developed by the PERSIAN (Prospective Epidemiological Research Studies in Iran) mega cohort study and was approved by the Ethics Committees in the Ministry of Health and Medical Education, the Digestive Diseases Research Institute, Tehran University of Medical Sciences, Iran. The details of this study have been published elsewhere [17, 18]. This cohort study was approved by the Ethics Committee of Kermanshah University of Medical Sciences (No: KUMS.REC.1394.318).

**Fig. 1:**
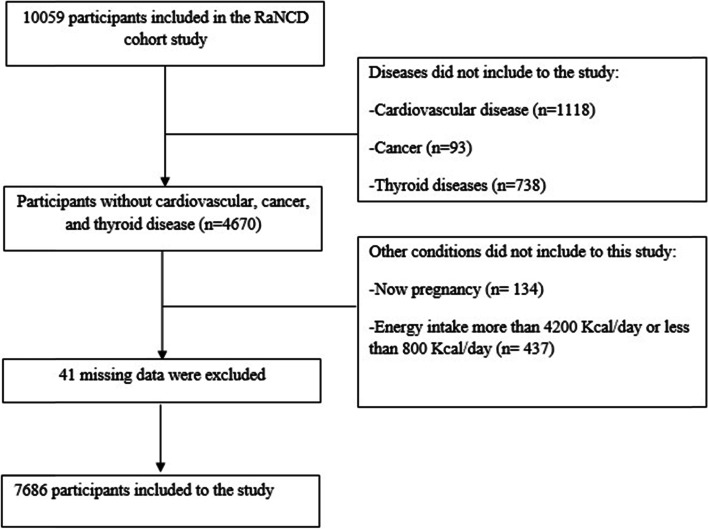
Flowchart of subjects’ selection

The inclusion criteria for this study were participants who provided complete information for the RaNCD cohort study. We also did not include participants with cardiovascular diseases (*n* = 1118), thyroid (*n* = 738), and cancer (*n* = 93) diseases due to possible dietary changes. Likewise, pregnant women (*n* = 134) were not included in this study. After excluding these participants, the participants whose calories intake was not in the range of 800–4200 Kcal/day (*n* = 437), were not included in the study. Furthermore, 41 participants with missing data were excluded. **(**Fig. 1**).**

### Data sources/ measurements

The necessary data were obtained from the RaNCD cohort study, including demographics (age and gender), physical activity, dietary intake, anthropometric indices, and medical history of diseases including CVDs, diabetes, thyroid diseases, cancer, and chronic LBP. The RaNCD physician assessed the medical history. History of smoking and drinking was also evaluated based on the participants’ history of smoking, being a passive smoker, and alcohol consumption. All the data were recorded in the RaNCD cohort study [17].

Anthropometry.

Participants’ weight was measured with InBody 770 device (Inbody Co, Seoul, Korea) with as little as clothing possible and without shoes in the study site in Ravansar. The automatic stadiometer BSM 370 (Biospace Co., Seoul, Korea) was applied to measure their height in a standing position without shoes with a precision of 0.1 cm. Body mass index (BMI) was calculated by dividing weight in kg into height square in meters. The non-stretched and flexible tape was used to measure waist circumference (WC) in a- standing position at the level of the iliac crest three times, and the average was recorded.

### Derivation of empirical dietary patterns

Participants’ diets were assessed using a valid, semi-quantitative 118-item food frequency questionnaire (FFQ) questionnaire developed for the RaNCD cohort study. The details of this questionnaire have been described in previous studies [17, 19]. 118 food items (in grams) were categorized into 31 food groups based on the nutrient content similarity to determine dietary patterns **(**Table 1**).** Principal component analysis was used to identify the major dietary patterns. The varimax rotation was applied to create a distinct and straightforward matrix in the factor analysis. The scree-plot was drawn to determine the number of matrix components (the major dietary patterns). We selected the first three major dietary patterns with eigenvalues greater than 1. Overall, the factor score for each dietary pattern was calculated by summing the food intakes of that group in terms of their factor loading, and each participant received a score for each pattern in terms of factor scores. Dietary pattern scores, i.e. factor scores, were divided into tertiles.

**Table 1:** Food groupings used in the dietary pattern analyses

Food groups	Dietary components
**Vegetables**	Cauliflower, lettuce, cucumber, onion, green bean, mushroom, pepper, garlic, turnip, eggplant, others
**Fresh fruits**	Melon, watermelon, honeydew melon, plums, prunes, apples, cherries, sour cherries, peaches, nectarine, pear, fig, date, grapes, kiwi, pomegranate, strawberry, banana, persimmon, berry, pineapple, oranges, others
**Dried fruits**	Dried apricots, Dried berries, raisins, and other type dried fruits
**Dairy**	Milk, yogurt, yogurt drink (doogh), cheese, chocolate milk, crud (Kashk), pizza cheese
**Tomato**	Tomato
**Carotene-rich vegetables**	Yellow squash, carrot
**Condiments**	Condiments
**Pickles**	Pickles
**Legumes**	All type beans, peas, lentils, mung bean, soy
**Whole grain**	Dark bread (Iranian), wheat, barley
**Starchy vegetables**	Corn, green peas, green squash
**Vegetable oil**	Vegetable oil
**Natural juices**	All fruit juices
**Butter**	Butter, margarine, mayonnaise
**Olive**	Olive and olive oil
**Organ meat**	Heart, kidney, liver, tongue, brain, offal
**Red meat**	Beef, lamb, minced meat
**Fish**	All fish types
**Processed meat**	Hamburger, sausage, delicatessen meat, meat pizza
**Soft drink**	Soft drink, Sugar sweetened beverage
**Nuts**	Almond, peanut, walnut, pistachio, hazelnut, seeds
**Egg**	Egg
**Poultry**	Chicken
**Snack**	Corn puffs, potato chips, French fries
**Sweets and desserts**	Cookies, cakes, biscuit, muffins, pies, chocolates, ice- cream, honey, jam, sugar cubes, sugar, candies, others
**Tea and coffee**	Tea and coffee
**Hydrogenated fat**	Hydrogenated fats, animal fats
**Salt**	Salt
**Potato**	Potato
**Refined grain**	White breads (lavash, baguettes), noodles, pasta, rice

### Physical activity

The physical activity level of the RaNCD participants was assessed using the standard questionnaire designed for PERSIAN Cohort. The questionnaire included 22 questions about the daily activity status. The responses were reported based on the metabolic equivalent of task per hour per day (MET/h/day). The detail of this questionnaire was described in the previous study [17].

### Outcome measurement

All participants completed self-reports about chronic LBP. The pain area was surveyed based on the RaNCD cohort study physician’s opinions and participants’ responses to her questions 1) Do you experience LBP that lasted more than a few months and interfered with their daily activities? In addition, has it lasted so far? (Yes/ No); 2) Do you have a history of back stiffness for more than an hour in the morning? (Yes/ No); 3) Do you have a history of arthralgia? (Yes/ No); 4) Do you have a history of joint stiffness for more than an hour in the morning? (Yes/ No). These questions were administered by the PERSIAN mega cohort study to evaluate chronic diseases in all Iranian adults ages≥35 years. Based on self-report and their medical history after physical examination by the physician, chronic LBP has been diagnosed the presence of LBP for a few months, which led to limited daily activities and had been sought for its treatment, such as medication, medical consultation, or physiotherapy. Furthermore, the physician did not consider pain associated with malignancies in the spinal cord area, infections, and fractures as chronic LBP [20].

### Statistical analysis

SPSS 20 (IBM Corp, Chicago, IL, USA) and Stata, version 14 (Stata Corp, College Station, TX) were applied for all statistical analysis. We reported quantitative variables by mean ± standard deviation (SD) and qualitative variables using frequency (%). Firstly, Dietary pattern scores, i.e. factor scores, were divided into tertiles. The comparison of participants’ baseline characteristics was evaluated using Chi-square and ANOVA tests based on the tertiles of all three dietary patterns. Binary logistic regression in crude and adjusted odds ratios (OR) and 95% confidence intervals (CI) was used to determine the association between chronic LBP and categories of three dietary patterns. In adjusted model 1, age (continuous), sex (categorical), smoking (categorical), and drinking (categorical) were adjusted. In adjusted model 2, we controlled the variables in model 1, diabetes (categorical), physical activity (continuous), body mass index (continuous), WC (continuous), energy intake (continuous), and treatment for chronic LBP (categorical). In all analyses, the first tertile of dietary patterns was considered as the reference category. Further, we considered a fractional polynomial plot for high protein, energy-dense diets concerning chronic LBP to illustrate this association better. *P*-values were considered significant at the level of < 0.05.

## Results

Seven thousand six hundred eighty-six of the RaNCD participants met the study inclusion criteria in the current study.51.3% of them were male. We found that 22.5% of the participants had chronic LBP. The factor analysis results introduced three dietary patterns with a factor loading of food groups of more than 0.2 **(**Table 2**).** The major dietary patterns were identified are as follows: 1) the vegetarian diet included vegetables, whole grains, legumes, nuts, olive, vegetable oil, fruits, and fruit juice; 2) the high protein diet related to higher adherence to red and white meat, legumes, nuts, and egg; and 3) the energy-dense diet characterized by a higher intake of salt, sweets, dessert, hydrogenated fat, soft drink, refined grains, tea, and coffee. Table 2 shows the rotated component matrix of each food groups and the correlation coefficient between each food group and dietary patterns.

**Table 2:** Factor loading of food groups in all dietary patterns

Food groups	Vegetarian dietary pattern	High protein dietary pattern	Energy dense diet
**Leafy vegetables**	.717	–	–
**Fresh fruits**	.630	.274	–
**Dried fruits**	.563	–	–
**Dairy**	.485	–	–
**Tomato**	.455	–	–
**Carotene-rich vegetables**	.439	.226	–
**Condiments**	.439	–	–
**Pickles**	.402	–	–
**Legumes**	.378	.345	–
**Whole grain**	.369	–	–
**Starchy vegetables**	.354	–	–
**Vegetable oil**	.330	–	−.248
**Natural juices**	.322	.239	–
**Butter**	.319	–	.276
**Olive**	.247	–	–
**Organ meat**	–	.611	–
**Read meat**	–	.578	–
**Fish**	–	.578	–
**Processed meat**	–	.516	–
**Soft drink**	–	.496	.295
**Nuts**	.360	.435	–
**Egg**	–	.330	.221
**Poultry**	–	.311	.209
**Snack**	–	.287	.206
**Sweets and desserts**	–	–	.738
**Tea and coffee**	–	–	.654
**Hydrogenated fat**	–	–	.500
**Salt**	–	–	.388
**Potato**	.251	–	.342
**Refined grain**	–	–	.331
**Variance %**	11.04	19.47	26.67

Values < 0.2 have been removed for clar

The highest tertiles of high protein and energy-dense diets were related to higher BMI and WC compared to the lowest tertile. (*P* < 0.001), while higher adherence to the vegetarian dietary patterns was significantly related to higher BMI and WC (*P* < 0.001) **(**Table 3**).**

**Table 3:** Baseline characteristics of studied participants

Variables	Total (*n* = 7686)	Vegetarian dietary pattern			P**	High protein dietary pattern			P**	Energy dense diet			P**
		T1 (*n* = 2562)	T2 (*n* = 2562)	T3 (*n* = 2562)		T1 (*n* = 2562)	T2 (*n* = 2562)	T3 (*n* = 2562)		T1 (*n* = 2562)	T2 (*n* = 2562)	T3 (*n* = 2562)	
Age (year)	47.28 ± 7.99^a^	47.56 ± 8.19	47.31 ± 8.04	46.98 ± 7.74	0.032	48.97 ± 8.11	47.04 ± 7.92	45.84 ± 7.63	< 0.001	47.45 ± 8.13	47.49 ± 7.98	46.91 ± 7.86	0.015
Weight (kg)	72.77 ± 13.69	70.24 ± 13.44	72.86 ± 13.6	75.23 ± 13.59	< 0.001	70.75 ± 13.44	72.16 ± 13.50	75.41 ± 13.72	< 0.001	72.33 ± 12.97	72.30 ± 13.47	73.69 ± 14.55	< 0.001
BMI (kg/m^2^)	27.28 ± 5.19	26.48 ± 4.51	27.33 ± 6.08	28.03 ± 4.72	< 0.001	27.66 ± 4.83	27.19 ± 6.10	26.98 ± 4.47	< 0.001	27.70 ± 6.01	27.13 ± 4.47	27 ± 4.94	< 0.001
WC (cm)	96.73 ± 10.67	95.42 ± 11.01	97.10 ± 10.37	97.66 ± 10.5	< 0.001	97.45 ± 11.16	96.46 ± 10.46	96.27 ± 10.35	< 0.001	97.44 ± 10.30	96.74 ± 10.21	96 ± 11.42	< 0.001
PA (MET hour/ day(	41.27 ± 8.38	41.46 ± 8.29	41.27 ± 8.49	41.06 ± 8.36	0.243	40.83 ± 7.46	40.99 ± 7.88	41.98 ± 9.60	< 0.001	39.36 ± 6.68	41.21 ± 8.12	43.23 ± 9.63	< 0.001
Sex, male, %	51.3	51.2	52.3	50.9	0.589	32.1	49.7	72.6	< 0.001	41	49.7	63.7	< 0.001
Drinking, %	6.6	6.7	6.9	6.3	0.689	2.3	5.7	12	< 0.001	4.5	5.7	9.8	< 0.001
Smoking, %	20.4	22.7	20.6	18.2	< 0.001	16.3	18.7	26.6	< 0.001	11.5	19.3	30.6	< 0.001
Diabetes, %	6.5	4.8	6.6	8.2	< 0.001	8.7	5.7	5.3	< 0.001	9.3	6.2	4.1	< 0.001
Chronic LBP, %	22.5	22.9	22.1	22.9	0.709	25.3	21.6	21.1	< 0.001	21.8	22	24.1	0.101

*BMI* Body mass index, *WC* Waist circumference, *PA* Physical activity, *LBP* low back pain

^a^Mean ± SD **P-values were obtained ANOVA and Chi square test

Our results showed that the mean of PA in all participants was 41.27 ± 8.38, in which the third tertiles of the high protein and energy-dense diet, the mean of PA was significantly higher than their first tertiles (*P* < 0.001) **(**Table 3**).** According to Table 3, diabetes was prevalent in 6.5% of the studied participants. In this study, the prevalence of chronic LBP decreased significantly with higher adherence to high protein dietary pattern (*P* < 0.001). However, this prevalence was not significantly different with higher following the two other major dietary patterns (vegetarian and energy-dense diet). Other characteristics of the studied participants are presented in Table 3.

Multivariable-adjusted odds ratios and 95% confidence intervals for chronic LBP across categories of three dietary patterns are indicated in Table 4**.** The highest tertile of the high protein dietary pattern was associated with lower odds of chronic LBP as compared to the lowest tertile (OR: 0.79, 95% CI: 0.69–0.9); such that after controlling for age, sex, smoking, drinking, diabetes, physical activity, body mass index, WC, energy intake and treatment this association remained (OR: 0.84, 95% CI: 0.72–0.97).

**Table 4:** Multivariable-adjusted odds ratios and 95% confidence intervals for chronic low back pain across categories of three dietary patterns

Major dietary pattern	Categories	Crude	Model 1^a^	Model 2^b^
Vegetarian dietary pattern	T1	1	1	1
	T2	0.95 (0.83–1.08)	0.96 (0.84–1.09)	0.94 (0.82–1.07)
	T3	0.99 (0.87–1.13)	1.01 (0.89–1.16)	0.96 (0.84–1.11)
P- trend		0.965	0.798	0.633
High protein dietary pattern	T1	1	1	1
	T2	0.81 (0.71–0.93)	0.86 (0.75–0.98)	0.85 (0.75–0.98)
	T3	0.79 (0.69–0.0.9)	0.88 (0.76–1.01)	0.84 (0.72–0.97)
P- trend		< 0.001	0.069	0.019
Energy dense diet	T1	1	1	1
	T2	1 (0.88–1.15)	1.1 (0.88–1.15)	1 (0.87–1.14)
	T3	1.13 (0.99–1.29)	1.16 (1.01–1.33)	1.13 (1.01–1.32)
P- trend		0.055	0.026	0.05

^a^Model 1 adjusted for age, sex, smoking, and drinking

^b^Model 2 adjusted for variables in model 1, diabetes, physical activity, body mass index, WC, energy intake, treatment

**Fig. 2:**
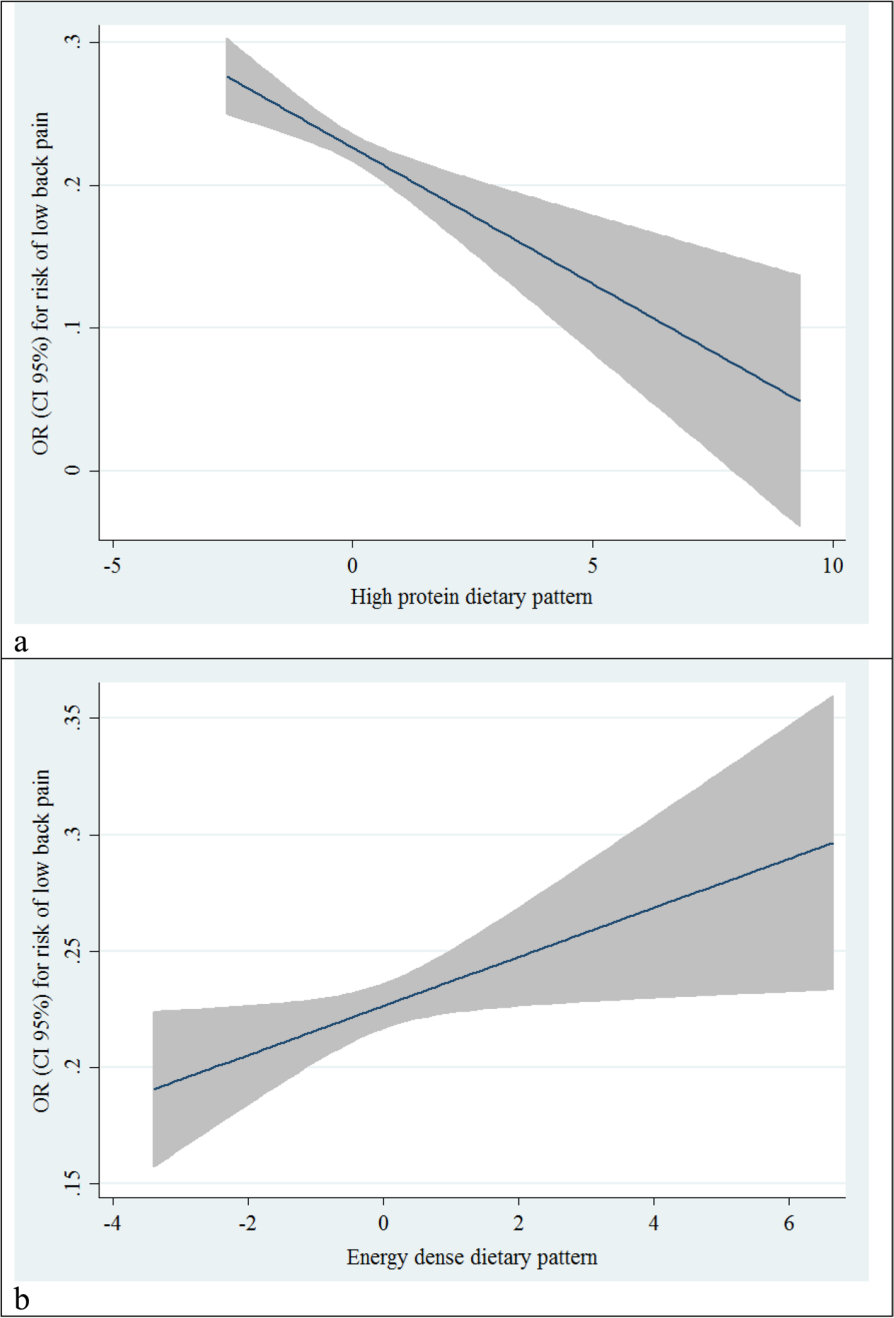
Liner regression odds ratios and 95% confidence intervals for chronic low back pain across categories of high protein (**a**) and energy dense diets (**b**)

In addition, after controlling for the mentioned potential confounders, The highest tertile of the energy-dense diet was associated with higher odds of chronic LBP than the lowest tertile (OR: 1.13, 95% CI: 1.01–1.32). Figure 2 shows odds ratios and 95% confidence intervals for chronic LBP across high protein and energy-dense diet categories.

However, no significant association was found between adherence to vegetarian dietary pattern and chronic LBP either before or after adjusting the confounders **(**Table 4**)**.

## Discussion

In the current study, we found that higher adherence to the high protein dietary pattern was inversely associated with chronic LBP, while odds of chronic LBP were increased with higher adherence to the energy-dense diet. LBP is a common pain experienced during adulthood, and it is believed that nutrition can affect the formation and severity of chronic LBP [21, 22]. Accordingly, the current study evaluated the relationship between major dietary patterns and chronic LBP.

In the current study, the prevalence of chronic LBP was significantly decreased with higher adherence to high protein dietary pattern. There was a significant association between high protein dietary pattern and chronic LBP. After controlling for potential confounders, participants in the third tertile of high protein dietary pattern were 12% lower odds of chronic LBP compared to participants in the lowest group. A randomized clinical trial by Kirk et al. [23] showed that dietary protein supplementation significantly improved skeletal muscle function. Another clinical trial by Shell et al. [24] showed that administration of amino acids precursors could improve chronic LBP and decrease the level of IL-6 and CRP. Nutritional mechanisms in the development of chronic LBP include affecting brain-gut axis neurotransmitters and changes in gut-derived neurotransmitters such as glutamate, which also affect the brain system and induce chronic pain [25]. Essential and semi-essential amino acids deficiency interfere with the production of neurotransmitter precursors that can affect pain sensation [24]. Other factors worsening chronic LBP include decreased muscle mass and some degree of sarcopenia [26, 27]. Skeletal muscle strength begins to decline in middle age in both men and women [28]. Adequate protein intake is one of the main factors in maintaining this muscle strength [29]. The type and amount of protein determine muscle mass’s effect [30, 31]. In this study, the high protein diet related to higher adherence to red and white meat, legumes, nuts, and egg involving protein with high biological value and essential micronutrients (e.g., calcium, iron, zinc, choline, vitamin B_12_) that are important for growth and development, developing of neurotransmitters, improving skeletal muscle mass and strength [32, 33].

Our study also found that adherence to the unhealthy diet was positively associated with chronic LBP. After adjusting the potential confounders, participants in the third tertile energy-dense diet were 15% higher odds of chronic LBP compared to participants in the lowest.

The unhealthy diet components in our study are most similar to the Western diet involving a higher intake of refined grains, red meat, processed meat, high saturated fat, trans-fatty acids, sweet sugary foods, and caffeine [34]. Following this dietary pattern was associated with an increased level of inflammatory markers such as IL-6 and CRP, leading to a decrease in pain threshold in chronic LBP [35–37]. Song et al. [38] reported that a high-fat diet was related to increased chronic LBP in the animal model. Another study was shown that higher adherence to sugary foods was decreased muscle strength (OR: 1.06 CI 95%: 1.01–1.12) [39]. Other studies also found that low-protein, high-sugar, high-fat diets were associated with more chronic LBP and higher CRP levels [9, 40–42]. Therefore, the unhealthy diet in this study was characterized by intake of salt, sweets, desserts, hydrogenated fat, soft drink, refined grain, tea, and coffee, which these dietary components can increase inflammation and consequence chronic LBP.

No significant relationship was found between the vegetarian diet and chronic LBP in this study. Although we controlled potential confounders, we did not observe any association between the vegetarian diet and chronic LBP. In fact, this dietary pattern contains high levels of essential antioxidants such as vitamin C, vitamin E, vitamin A, and all carotenoids [43, 44]. These antioxidants have anti-inflammatory effects and can reduce the pain threshold in these patients [45]. On the other hand, vegetable-based diets produce short-chain fatty acids that stabilize the beneficial intestinal microbiome, and substances derived from this microbiome environment can affect the brain-gut system and reduce systemic and central inflammation [46]. Furthermore, this vegetable diet can relieve musculoskeletal pain [47]. In the present study, the vegetarian dietary pattern was related to the intake of vegetables, whole grains, legumes, nuts, olive, vegetable oil, fruits, and fruit juice. The intake of these food groups in participants with and without chronic LBP seems to be the same and we could not find any association.

## Limitations

This is the first study to evaluate the relationship between major dietary patterns and chronic LBP among the Kurdish population; however, this study suffered from some limitations. Firstly, this is a cross-sectional study and the cause-and-effect relationship was unclear. Second, dietary intake was assessed by FFQ, and the error of recalling food intake should not be ignored. However, the questionnaire was presented by trained nutritionists. In addition, the degree and severity of chronic LBP in the RaNCD cohort study were not measured. Therefore, further studies are recommended without these limitations.

## Conclusions

According to the findings of this study, higher adherence to high protein diet had protective effects on chronic LBP prevalence. In addition, we found that following energy dense diet was associated with higher odds of chronic LBP. Therefore, it is recommended that people prone to chronic LBP consider a high biological value protein in their daily diet, and reduce their intake of salt, sweets, dessert, hydrogenated fat, soft drink, refined grain, tea, and coffee.

## Data Availability

Data will be available upon request from the corresponding author.

## Abbreviations

LBP: Low back pain
CRP: C- reaction protein
TNF-α: tumor necrosis factor alpha
RaNCD: Ravansar non- communicable diseases
BMI: Body mass index
OR: Odds ratios
CI: Confidence intervals
